# Combined and mediating effects of remnant cholesterol and renal function on hypertension risk in Chinese middle-aged and elderly people

**DOI:** 10.3389/fendo.2025.1442918

**Published:** 2025-02-13

**Authors:** Mengjie Zhao, Mengli Xiao, Huie Zhang, Qin Tan, Jinjin Ji, Yurong Cheng, Fang Lu

**Affiliations:** ^1^ Xiyuan Hospital, China Academy of Chinese Medicine Sciences, Beijing, China; ^2^ Graduate School of China Academy of Chinese Medical Sciences, Beijing, China; ^3^ Graduate School of Beijing University of Chinese Medicine, Beijing, China; ^4^ National Medical Products Administration (NMPA) Key Laboratory for Clinical Research and Evaluation of Traditional Chinese Medicine, Beijing, China; ^5^ National Clinical Research Center for Chinese Medicine Cardiology, Beijing, China

**Keywords:** remnant cholesterol, renal function, estimated glomerular filtration rate, hypertension, mediation analysis

## Abstract

**Background:**

Emerging evidence indicates a potential correlation between remnant cholesterol (RC) and the development of vascular damage and hypertension. Nevertheless, the precise relationship between RC and hypertension in relation to renal function remains uncertain. The objective of this investigation was to employ a cohort design to evaluate the intricate correlation between RC and renal function in relation to hypertension.

**Methods:**

The present investigation utilized data from the China Health and Retirement Longitudinal Study (CHARLS), encompassing a total of 5,109 participants, for comprehensive data analysis and examination. Cox regression analysis was employed to examine the interplay among RC, renal function, and hypertension within the context of this research study. This study utilized restricted cubic spline (RCS) analysis to elucidate the interaction between RC, renal function, and hypertension, specifically examining the mediating role of renal function in the RC-hypertension nexus. Furthermore, we employed mediation analysis to investigate the potential mediating role of renal function in the association between RC and hypertension.

**Results:**

After a 9-year follow-up period, the incidence of hypertension in the population under investigation was observed to be 19.01%. The Kaplan-Meier curves demonstrated a notable and statistically significant elevation in the prevalence of hypertension within the subgroup characterized by higher RC and impaired renal function (P <0.001). However, in Cox regression analyses, the risk of developing hypertension was significantly higher (*P <*0.05) in those with high RC and high estimated glomerular filtration rate (eGFR), and those with high RC and low eGFR, compared with those with low RC and high eGFR, after adjusting for confounders. The analysis of RCS demonstrated a significant positive linear correlation between baseline RC and the prevalence of hypertension. Additionally, there was a notable negative linear correlation observed between eGFR levels and the prevalence of hypertension. RC and eGFR did not interact with any of the subgroup variables. eGFR lowering mediated 6% of the associations between RC and hypertension.

**Conclusion:**

The findings of this study unveiled a substantial correlation between elevated RC, diminished eGFR levels, and the risk of developing hypertension. In addition, renal function may mediate the correlation between RC and hypertension risk.

## Introduction

In recent years, there has been an escalating prevalence of both hypertension and chronic kidney disease (CKD), and there is an interconnection between these two diseases, which seriously increases the socio-economic burden (1–4). Hypertension is recognized as a risk factor for renal insufficiency (5–8). Numerous studies have demonstrated the substantial influence of renal injury on the progression of hypertension (9, 10). Similar results have been observed in children with low birth weight (11). Experimental animal studies have demonstrated that even minor renal impairment can induce hypertension in rats (12). However, there is still controversy about the association between kidney injury and hypertension (13–15). This was verified by the latest Mendelian randomization study, which found that improved renal function only had a causal effect on lowering blood pressure, while the reverse causality between the two was not significant (16).

Residual cholesterol (RC) is the cholesterol component of triacylglycerol-rich lipoproteins (17). Over the past few years, extensive research has revealed the pivotal contribution of RC and low-density lipoprotein (LDL) to the pathogenesis and advancement of hypertension (18–20). Recent studies have shown that the association between hypertension and RC exceeds that of LDL in American adults (21). Patients with CKD are often associated with dyslipidemia (22–25), which is manifested by elevated triacylglycerol (TG) levels, reduced concentration and function of high-density lipoproteins (HDL), and elevated levels of small dense low-density lipoproteins (sdLDL), which have atherosclerotic effects (26). An abundance of studies has consistently demonstrated a robust correlation between elevated RC levels and the onset of CKD (27–29). Based on a recent observational study, a positive correlation has been observed between the levels of RC in individuals with hypertension and their susceptibility to developing CKD (30). However, the intricate interplay among RC levels, renal function, and hypertension remains inadequately elucidated in the existing literature.

Therefore, the present investigation endeavors to undertake a comprehensive, large-scale, long-term follow-up study, with the goal of furnishing additional evidence pertaining to the intricate relationships linking RC levels, renal function, and hypertension.

## Methods

The dataset employed in this study was sourced from the comprehensive China Health and Retirement Longitudinal Study (CHARLS) database. The CHARLS national baseline survey began in 2011, with follow-up assessments every 2 years, and five rounds of the survey have been completed to date (2011, 2013, 2015, 2018, 2020). Conducted via the face-to-face interview method, the CHARLS is a research survey that primarily focuses on individuals and families aged 45 years and above within China’s middle-aged and older population. The study also incorporates physical measurements during follow-up visits (31). For the utilization of data in this study, formal authorization was sought from the CHARLS project team, and ethical approval was granted by the Ethics Review Board of Peking University (No. IRB00001052-11015) (32).

**Figure 1:**
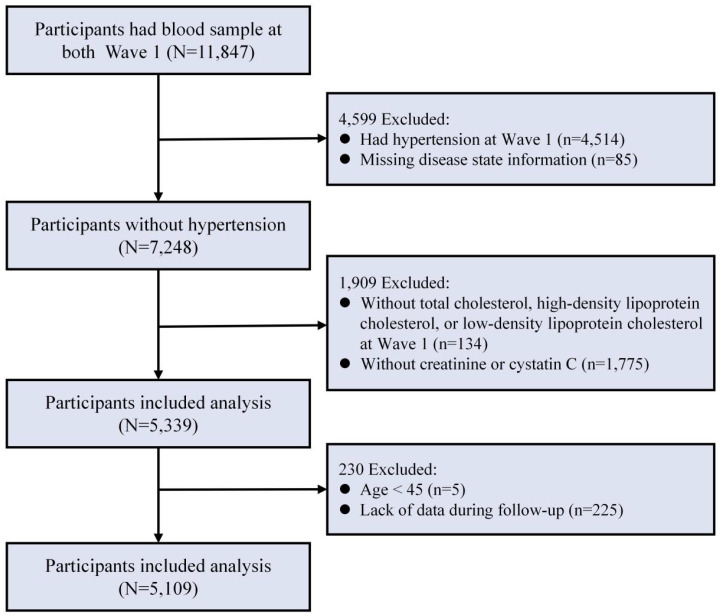
Flowchart for screening of research subjects.

This study was analyzed using follow-up data from 2011-2020. Inclusion criteria were as follows: (1) age ≥45 years at wave 1; (2) had total cholesterol (TC), HDL, LDL, creatinine (Cr), and cystatin C (Cys C) at wave 1; (3) not yet hypertensive at wave 1; and (4) hypertension status information recorded at follow-up. The study population encompassed a comprehensive cohort of 5,109 participants. The screening process for participants is illustrated in **Figure 1**.

### Data collection and definitions

All CHARLS projects collected demographic characteristics and lifestyle behaviors of the observed subjects in the form of questionnaires and tested blood biochemical indicators. The study encompassed the collection of the following variables. The demographic characteristics assessed in this study encompassed age, gender, educational attainment, and marital status. The calculation of body mass index (BMI) was based on the parameters of weight and height. Chronic disease status included dyslipidemia, heart disease, and stroke. Dyslipidemia was diagnosed by a self-reported physician-diagnosed, and/or current use of lipid-lowering drugs, and/or TC ≥ 240 mg/dl, TG ≥ 200 mg/dl, HDL-C < 40 mg/dl, LDL-C  ≥ 160 mg/dl (33). Medication history included lipid-lowering and hypoglycemic medications. Laboratory tests included blood biochemistry for TC, HDL, LDL, Cr, Cys C, hemoglobin A1c (HbA1c), and C-reactive protein (CRP).

### RC and eGFR

The RC value was calculated based on the observed concentrations of TC, LDL, and HDL. The formula for RC is: RC = TC-(LDL+HDL) (34, 35). The eGFR level was determined using the formula provided by the 2021 Chronic Kidney Disease Epidemiology Collaboration (CKD-EPI) (36). Impaired renal function was defined as an eGFR below 60 ml/min/1.73 m^2^ (37).

### Definition of hypertension

The primary outcome of this study was the occurrence of hypertension over the follow-up period, spanning from wave 2 to wave 5. Hypertension was specifically identified as the self-reported presence of hypertension, a systolic blood pressure (SBP) equal to or greater than 140 mmHg, and/or a diastolic blood pressure (DBP) equal to or greater than 90 mmHg as measured during follow-up, or current treatment with antihypertensive medication (38).

### Statistical analysis

Due to the non-normal distribution of the data, the baseline characteristics of the study population were statistically presented using medians along with their interquartile ranges (IQRs) and percentages, respectively. In order to assess the correlation between RC levels, eGFR, and the likelihood of developing hypertension, we employed multivariable-adjusted Cox regression models. RC levels were categorized into two groups according to the median. After calculating eGFR values for each participant, we segregated the population into two distinct groups, utilizing a cutoff value of 60. Then, RC and eGFR were combined to group the population into four categories. In addition, combined grouping based on RC levels (using tertiles) and eGFR values (60 and 90) divided the participants into 9 categories. To investigate the potential dose-response association between RC levels, eGFR, and the risk of developing hypertension, we employed a combination of restricted cubic spline (RCS) analysis and Cox regression modeling.

Furthermore, additional subgroup analyses were conducted. To strengthen the reliability of our results, we performed a sensitivity analysis utilizing the inverse probability of treatment weighting (IPTW) technique. IPTW is an individual standardization method through propensity score weighting. Using the principle of the standardization method, the reciprocal of the propensity score value is taken to obtain the weight, and each study object is weighted to generate a larger sample. The weighted sample can be seen as the exposure is randomly assigned and can eliminate the influence of confounders (39, 40). In addition, we validated the analyses separately by removing missing data, excluding patients with cardiovascular disease (CVD) at baseline, excluding patients with a history of taking lipid-lowering and glucose-lowering medications, and additionally adjusting for uric acid (UA), etc. To further assess the stability of the Cox regression model, we calculated the E-values to evaluate the minimum strength of association that an unmeasured confounder would need to have with both RC and eGFR in relation to hypertension to completely nullify the observed association.

We constructed and analyzed two sets of mediation models. In the first mediation model, we assessed the mediating role of eGFR levels between RC and hypertension using hypertension as the dependent variable, RC as the independent variable, and eGFR as the mediating variable. In the other model, we examined the mediating role of RC levels between eGFR and hypertension. Each model was analyzed separately according to unadjusted versus adjusted confounders. Sampling was repeated 5000 times using a nonparametric bootstrap method to assess the indirect effects of RC and eGFR (41). The Bootstrap method is a nonparametric resampling procedure. In this study, Bootstrap samples similar to the original samples were obtained by performing 5,000 repetitive samples with put-backs on the existing samples. We computed the total and indirect effects of the samples collected during each sampling stage, allowing us to derive the 95%CI for the mediating effect. A significant mediating effect was identified if the confidence interval excluded zero.

The joint effects analysis estimated the joint effect of RC combined with eGFR on incident hypertension by testing additive and multiplicative interactions in a multivariate corrected Cox model. Binary RC and binary eGFR were analyzed by including them in multivariate Cox regression models as product terms and adjusting for other possible confounders, with HRs reflecting multiplicative interactions (42).

The evaluation of additive interaction is based on three indicators, namely (1) the relative excess risk due to interaction (RERI), (2) the attributable proportion due to interaction (AP), and (3) the synergy index (SI). The RERI is used to measure the magnitude of the relative effect due to the attributable interaction effect. The more significant the absolute value of RERI, the stronger the interaction between the factors. The AP indicates the proportion of the attributable interaction effect in the joint effect of the two factors. The larger the absolute value of the AP, the stronger the interaction between the factors. SI refers to the ratio of the effect of the simultaneous presence of the two factors to the sum of the effects of the two factors alone. The larger the absolute value of SI, the stronger the interaction between the factors. In general, synergistic interactions existed when both RERI and AP were >0, the CI did not include 0, SI was >1, and the confidence interval did not include 1. When RERI and AP are <0, the confidence interval does not include 0, SI <1 and the confidence interval does not include 1, there is an antagonistic interaction (43). The delta method was employed to determine the 95% confidence intervals for the three indicators in question (44).

We performed the statistical analysis using Stata 16.0 and R 4.1.3 software, and considered a significance level of P < 0.05 to indicate statistical significance.

## Results

### Baseline characteristics

There were 5,109 participants in this study, with a median age of 66. Males comprised 47.39% of the participants, 68.47% had primary education or higher, and 88.37% were married or cohabiting (**Table 1**). The median RC at baseline was 18.94 mg/dL. Compared with the remaining three groups, those with high RC (≥ 18.94) and impaired renal function had a higher proportion of obesity, comorbid diabetes mellitus, and use of lipid-lowering and glucose-lowering medications (*P <*0.05). Furthermore, elevated RC levels and reduced renal function showed a significant positive correlation with CRP, HbA1c, TC, and LDL levels, while demonstrating a negative association with HDL levels (P <0.05). During up to 9.0 years of follow-up, 971 (19.01%) individuals had hypertension.

**Table 1:** Characteristics of 5,109 participants categorized by RC and eGFR levels.

	Total	RC<18.94&eGFR≥60	RC<18.94&eGFR<60	RC≥18.94&eGFR≥60	RC≥18.94&eGFR<60	*P*
Age, years	66.00 (59.00, 74.00)	66.00 (59.00, 75.00)	82.00 (77.00, 86.00)	66.00 (59.00, 73.00)	81.00 (71.75, 88.00)	<0.001
Sex, n (%)						<0.001
Male	2421 (47.39)	1212 (48.58)	28 (84.85)	1156 (45.57)	25 (56.82)	
Education level, n (%)						0.091
No formal education	2398 (46.94)	1187 (47.58)	15 (45.45)	1172 (46.2)	24 (54.55)	
Primary school	1100 (21.53)	526 (21.08)	9 (27.27)	553 (21.80)	12 (27.27)	
Middle or high school	1419 (27.77)	685 (27.45)	5 (15.15)	723 (28.50)	6 (13.64)	
College or above	192 (3.76)	97 (3.89)	4 (12.12)	89 (3.51)	2 (4.55)	
Marital status, n (%)						0.054
Married	4515 (88.37)	2192 (87.86)	28 (84.85)	2261 (89.12)	34 (77.27)	
Other	594 (11.63)	303 (12.14)	5 (15.15)	276 (10.88)	10 (22.73)	
BMI, kg/m^2^, n (%)						<0.001
18.5-24	2939 (57.53)	1558 (62.44)	26 (78.79)	1330 (52.42)	25 (56.82)	
<18.5	460 (9.00)	275 (11.02)	4 (12.12)	176 (6.94)	5 (11.36)	
24-28	1305 (25.54)	538 (21.56)	2 (6.06)	758 (29.88)	7 (15.91)	
>28	405 (7.93)	124 (4.97)	1 (3.03)	273 (10.76)	7 (15.91)	
Dyslipidemia, n (%)						<0.001
Yes	1901 (37.21)	535 (21.44)	14 (42.42)	1323 (52.15)	29 (65.91)	
Diabetes, n (%)						<0.001
Yes	711 (13.92)	242 (9.70)	6 (18.18)	451 (17.78)	12 (27.27)	
Heart disease, n (%)						0.289
Yes	444 (8.69)	204 (8.18)	4 (12.12)	230 (9.07)	6 (13.64)	
Stroke, n (%)						0.579
Yes	60 (1.17)	32 (1.28)	0 (0)	27 (1.06)	1 (2.27)	
Lipid-lowering drugs, n (%)						0.003
Yes	125 (2.45)	42 (1.68)	1 (3.03)	80 (3.15)	2 (4.55)	
Hypoglycemic drugs, n (%)						0.036
Yes	110 (2.15)	49 (1.96)	1 (3.03)	56 (2.21)	4 (9.09)	
Smoking status, n (%)						0.045
Yes	2023 (39.60)	983 (39.40)	21 (63.64)	1001 (39.46)	18 (40.91)	
Drinking status, n (%)						0.266
Yes	1085 (21.24)	557 (22.32)	5 (15.15)	514 (20.26)	9 (20.45)	
CRP, mg/L	0.89 (0.50, 1.88)	0.80 (0.47, 1.72)	1.39 (0.96, 3.34)	0.97 (0.52, 1.99)	1.42 (0.82, 3.77)	<0.001
Hba1c, %	5.10 (4.90, 5.40)	5.10 (4.80, 5.40)	5.20 (4.80, 5.70)	5.10 (4.90, 5.40)	5.20 (5.00, 5.60)	<0.001
Cys C, mg/L	0.96 (0.84, 1.10)	0.98 (0.86, 1.11)	1.66 (1.39, 1.83)	0.93 (0.81, 1.07)	1.44 (1.26, 1.72)	<0.001
eGFR, ml/min/1.73 m^2^	104.10 (92.72, 112.50)	104.80 (94.26, 112.90)	53.41 (46.73, 56.31)	103.80 (92.58, 112.40)	54.76 (50.25, 57.30)	<0.001
SCr, mg/L	0.75 (0.64, 0.87)	0.73 (0.63, 0.85)	1.31 (1.27, 1.46)	0.75 (0.64, 0.87)	1.28 (1.17, 1.36)	<0.001
TC, mg/dL	187.90 (165.10, 213.00)	181.70 (160.80, 205.30)	179.00 (156.20, 207.20)	194.10 (170.90, 220.40)	207.80 (179.50, 237.80)	<0.001
HDL, mg/dL	50.64 (41.37, 61.08)	55.28 (47.55, 64.95)	53.74 (44.85, 58.38)	45.23 (37.11, 55.28)	47.36 (36.15, 60.31)	<0.001
LDL, mg/dL	113.70 (92.40, 135.30)	114.00 (94.33, 135.30)	111.30 (92.40, 151.50)	112.90 (90.46, 135.30)	124.30 (96.75, 143.90)	0.099
RC, mg/dL	18.94 (11.21, 30.54)	11.21 (7.34, 14.69)	11.98 (9.66, 14.30)	30.15 (23.97, 44.46)	28.61 (24.65, 42.91)	<0.001

### Association of RC and eGFR with risk of hypertension

**Figure 2:**
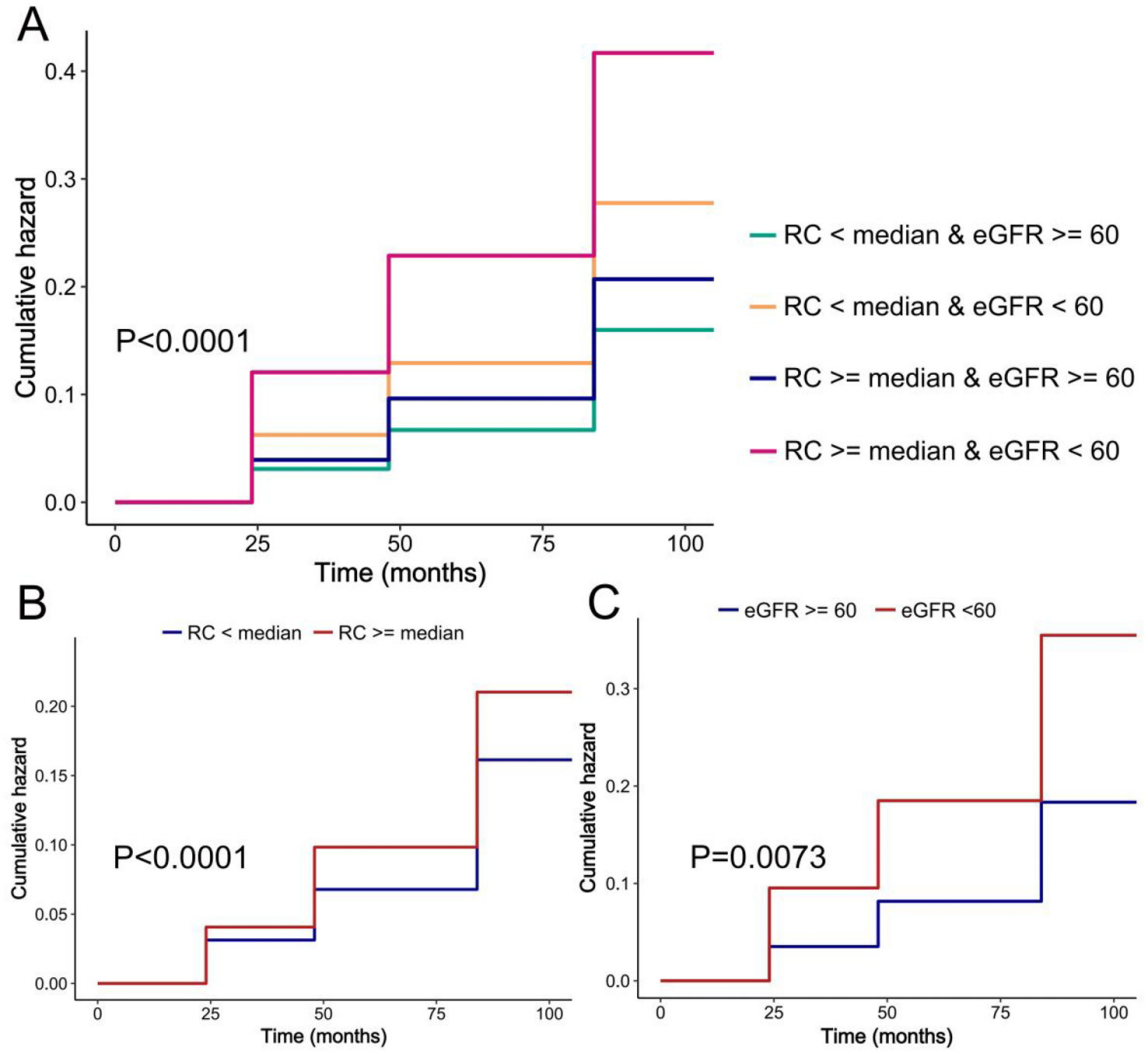
K-M plot of hypertension by RC and eGFR level. **(A)** RC and eGFR; **(B)** RC; **(C)** eGFR.

We evaluated the cumulative occurrence of hypertension by analyzing the combined baseline RC and eGFR values. Notably, individuals with elevated RC and compromised renal function exhibited the highest prevalence of hypertension, as depicted in **Figure 2A**. In addition, when both were assessed separately, we found a higher incidence of hypertension in either those with higher RC or those with impaired renal function, respectively (**Figures 2B, C**).

**Figure 3:**
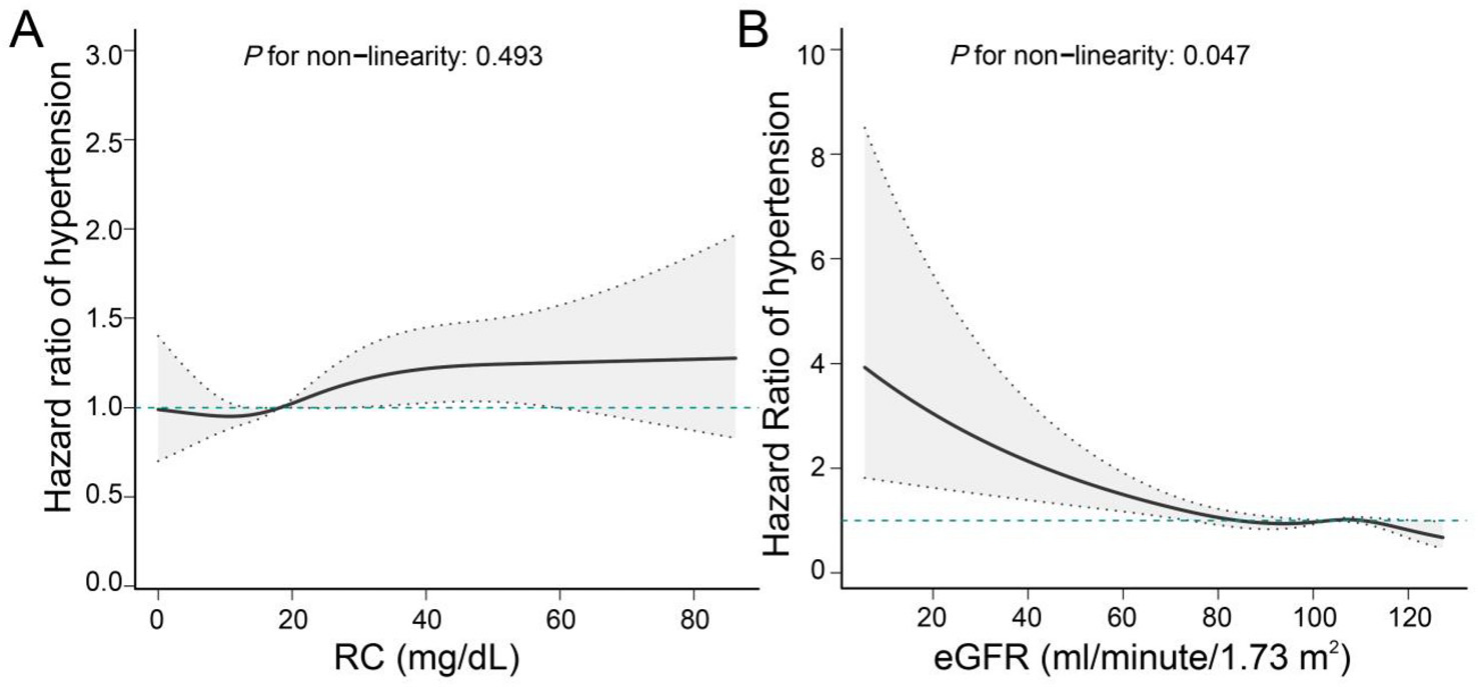
Linear associations of RC and eGFR with hypertension. **(A)** Association of RC with hypertension; **(B)** Association of eGFR with hypertension. We adjusted for potential confounders, including age, sex, education level, marital status, BMI, dyslipidemia, heart disease, stroke, smoking status, drinking status, lipid-lowering drugs, hypoglycemic drug, HbA1c, and CRP.

Upon adjustment for potential confounding factors, our analysis revealed a statistically significant elevation in the risk of hypertension among populations characterized by high RC and either high or low eGFR values, when compared to those with low RC and high eGFR values. The hazard ratios (HR [95%CI]) obtained were 1.15 [1.00, 1.31] and 2.10 [1.25, 3.54], as presented in **Table 2**. In comparison to the low RC group, the high RC population exhibited a heightened likelihood of developing hypertension, with a hazard ratio (HR [95%CI]) of 1.15 (1.01,1.32). The study revealed that individuals with impaired renal function faced a notably higher risk of hypertension compared to those with normal renal function (HR [95%CI]: 1.76 [1.16,2.67]), as demonstrated in **Table 2**. Our investigation unveiled a positive association between RC and the incidence of hypertension, whereas eGFR exhibited a negative correlation with the likelihood of developing hypertension (**Figure 3**).

**Table 2:** Cox regression to assess the association of RC and eGFR with hypertension.

	Crude	Model 1	Model 2	Model 3
	HR (95%CI)	HR (95%CI)	HR (95%CI)	HR (95%CI)
RC < 18.94 & eGFR ≥ 60	1(Ref)	1(Ref)	1(Ref)	1(Ref)
RC < 18.94 & eGFR < 60	1.55 (0.77, 3.13)	1.52 (0.76, 3.07)	1.58 (0.78, 3.20)	1.60 (0.79, 3.24)
RC ≥ 18.94 & eGFR ≥ 60	1.29 (1.14, 1.47)	1.30 (1.14, 1.47)	1.16 (1.01, 1.32)	1.15 (1.00, 1.31)
RC ≥ 18.94 & eGFR < 60	2.40 (1.43, 4.01)	2.31 (1.38, 3.87)	2.11 (1.25, 3.55)	2.10 (1.25, 3.54)
RC < 18.94	1(Ref)	1(Ref)	1(Ref)	1(Ref)
RC ≥ 18.94	1.30 (1.15, 1.48)	1.30 (1.15, 1.48)	1.16 (1.01, 1.33)	1.15 (1.01, 1.32)
eGFR ≥ 60	1(Ref)	1(Ref)	1(Ref)	1(Ref)
eGFR < 60	1.76 (1.16, 2.66)	1.71 (1.13, 2.6)	1.74 (1.15, 2.65)	1.76 (1.16, 2.67)

HR, hazard ratio; CI, confidence interval; eGFR, estimated glomerular filtration rate; RC, remnant cholesterol.

Median RC index: 18.94.

Model 1: age + sex + education level + marital status;

Model 2: Model 1 + BMI + dyslipidemia + heart disease + stroke + smoking status + drinking status;

Model 3: Model 2 + lipid-lowering drugs + hypoglycemic drug + HbA1c + CRP.

### Subgroup analysis

After stratifying for sex, age, education level, and BMI, we found no interaction between RC combined with eGFR and any of the subgroup variables (**Table 3**). Among participants aged ≥60 years, women, elementary school education or less, and overweight, we still found a higher risk of hypertension in those with high RC and low eGFR (*P <*0.05) (**Table 3**).

**Table 3:** Subgroup analysis of the association of the RC and eGFR with the risk of hypertension.

Subgroup	N	RC < 18.94 & eGFR ≥ 60	RC < 18.94 & eGFR < 60	RC ≥ 18.94 & eGFR ≥ 60	RC ≥ 18.94 & eGFR < 60	*P* interaction
Age, years						0.128
<60	1,333	1(Ref)	9.26 (2.21, 38.7)	1.47 (1.1, 1.96)	2.31 (0.54, 9.94)	
≥60	3,776	1(Ref)	1.21 (0.54, 2.73)	1.06 (0.91, 1.24)	1.98 (1.13, 3.47)	
Sex						0.564
Male	2,421	1(Ref)	1.71 (0.8, 3.65)	1.19 (0.97, 1.46)	1.52 (0.71, 3.28)	
Female	2,688	1(Ref)	1.21 (0.17, 8.79)	1.12 (0.93, 1.35)	3.14 (1.53, 6.42)	
Education level						0.961
Primary and below	3,498	1(Ref)	1.55 (0.69, 3.51)	1.17 (1, 1.37)	2.21 (1.26, 3.89)	
Secondary and above	1,611	1(Ref)	2.24 (0.55, 9.17)	1.1 (0.84, 1.43)	1.75 (0.43, 7.15)	
BMI, kg/m^2^						0.674
<24	3,399	1(Ref)	1.56 (0.73, 3.33)	1.07 (0.89, 1.27)	1.68 (0.83, 3.42)	
≥24	1,710	1(Ref)	1.84 (0.25, 13.25)	1.28 (1.03, 1.6)	3.17 (1.45, 6.94)	
Dyslipidemia						0.625
Yes	1,901	1(Ref)	1.16 (0.36, 3.72)	1.27 (1.01, 1.60)	2.36 (1.25, 4.45)	
No	3,208	1(Ref)	1.93 (0.79, 4.71)	1.07 (0.89, 1.27)	1.63 (0.61, 4.40)	
Heart disease						0.812
Yes	444	1(Ref)	1.62 (0.21, 12.34)	0.95 (0.61, 1.47)	2.01 (0.40, 10.17)	
No	4,665	1(Ref)	1.60 (0.75, 3.39)	1.16 (1.01, 1.34)	2.13 (1.22, 3.72)	
Stroke						0.403
Yes	60	1(Ref)	–	0.91 (0.19, 4.36)	–	
No	5,049	1(Ref)	1.54 (0.76, 3.12)	1.12 (0.98, 1.29)	1.94 (1.13, 3.33)	

In multivariate models, potential confounders other than grouping variables were adjusted for, including age, sex, education level, marital status, BMI, dyslipidemia, heart disease, stroke, smoking status, drinking status, lipid-lowering drugs, hypoglycemic drug, HbA1c, and CRP.

### Interaction of RC and eGFR with incident hypertension

Using RC, eGFR and the product term of the two as independent variables and new-onset hypertensive events as dependent variables, after controlling for a variety of confounders, cox regression showed that the HR and 95% CI for RC*eGFR was 1.15 (0.48,2.73), suggesting that there was no multiplicative interaction of RC and eGFR in hypertensive events (P >0.05) (**Table 4**). The evaluation indexes for the additive interaction of RC and eGFR were calculated and showed that RERI=0.36 (95% CI: -1.19, 1.90), AP=0.17 (95% CI: -0.51, 0.85), and SI=1.48 (95% CI: 0.25, 8.81). Thus, there was also no additive interaction between the two.

**Table 4:** Interaction of RC and eGFR on hypertensive events.

Interactive items	Interactive effects (95% CI)		
	Model 1	Model 2	Model 3
Additive effects			
RERI	0.49 (-1.09,2.07)	0.37 (-1.17,1.91)	0.36 (-1.19,1.90)
AP	0.21 (-0.40,0.82)	0.18 (-0.49,0.85)	0.17 (-0.51,0.85)
SI	1.60 (0.33,7.77)	1.50 (0.25,8.95)	1.48 (0.25,8.81)
Multiplicative effect	1.17 (0.49,2.79)	1.15 (0.48,2.75)	1.15 (0.48,2.73)

CI, confidence interval; RERI, relative excess risk due to interaction; AP, proportion attributable to interaction; SI, synergy index.

Model 1: age + sex + education level + marital status;

Model 2: Model 1 + BMI + dyslipidemia + heart disease + stroke + smoking status + drinking status;

Model 3: Model 2 + lipid-lowering drugs + hypoglycemic drug + HbA1c + CRP.

### Sensitivity analysis

In the sensitivity analysis, the E-value was 2.72, indicating that to explain the observed HR of 2.10, an unmeasured confounder would need to be associated with both RC and eGFR in relation to hypertension with an HR of at least 2.72, higher than and beyond the measured confounders (**Supplementary Figure 1**). In addition, we obtained consistent findings in several sensitivity analyses. The risk of hypertension prevalence was nearly identical to the above results when the model was constructed using IPTW, excluding those with CVD, those on lipid-lowering and glucose-lowering medications, and making additional adjustments for UA levels (**Supplementary Figure 2**). After deleting missing values, we found that the effect size was attenuated for high RC with high eGFR (*P >*0.05), although the effect size was increased for the high RC with low eGFR population (HR [95%CI]: 3.88 [1.52,5.47]) (**Supplementary Table 1**). When stratified by tertiles of RC and by 60 and 90 eGFR, compared with the other 8 groups, the highest risk of hypertension (HR [95%CI]: 1.97 [1.04,3.73]) was still observed in those with high RC (T3) and low eGFR (<60) (**Supplementary Table 2**).

The study utilized mediation analysis to investigate the relationship between RC, eGFR, and new-onset hypertension. The results showed that the mediation rate of eGFR was 3.14% in the unadjusted model. After adjusting for age, sex, education level, marital status, BMI, dyslipidemia, heart disease, stroke, smoking status, drinking status, lipid-lowering drugs, hypoglycemic drugs, HbA1c, and CRP, the proportion of mediators of eGFR was elevated (Adjusted for 6.00% vs. 3.14%) (**Supplementary Figures 3A, B**). Furthermore, in the unadjusted model, we observed a mediating role of RC between eGFR and new-onset hypertension. However, this mediating effect was attenuated after adjustment for confounders (*P >*0.05) (**Supplementary Figures 3C, D**).

## Discussion

This study enrolled a total of 5,109 Chinese adults aged 45 years or above. Following a follow-up period of 9.0 years, the study revealed a positive correlation between higher baseline RC indices and lower eGFR values with an elevated risk of developing hypertension. This association was found to persist by adjusting for known risk factors and IPTW procedures. The RCS curve visualizes the relationship between RC levels or eGFR values and hypertension. Furthermore, the findings of this study elucidated that a reduction in eGFR values partially mediated the association between RC indices and hypertension.

Several studies have provided evidence supporting the utility of RC, a novel lipoprotein measure, in predicting the likelihood of developing hypertension. A large cohort study of 24,252 Chinese adults found a high correlation between RC and hypertension risk. In addition, it was confirmed that an increase in RC preceded the development of hypertension using cross-lag analysis (20). In a separate cohort study conducted within a Chinese community, it was demonstrated that heightened RC levels exhibited a robust correlation with elevated central SBP (45). In an investigation using the U.S. database, it was observed that RC exhibited significant associations with hypertension, type 2 diabetes, and their co-morbid risks, potentially mediated by inflammatory responses (46). Considering the high prevalence of hypertension globally, even a small effect size of RC on hypertension (HR=1.15), when applied to millions of susceptible individuals, could have a significant impact on medical resource demands and disease burden. A prospective investigation involving middle-aged and elderly Japanese subjects revealed a positive correlation between RC levels and hypertension outcomes (47). A comprehensive lipid study conducted in Finland, encompassing a large sample size, demonstrated significant associations between several serum lipids, such as LDL, RC, and apolipoprotein B, and increased blood pressure. These findings were further validated through an inverse variance weighted fixed effects meta-analysis (18). It has also been reported that the RC index may increase the progression of CVD or adverse events in hypertensive patients (48).

It is worth noting that there may exist a connection between RC and impaired kidney function. According to a research study conducted by the Risk Assessment for Cancer in Chinese Patients with Diabetes Mellitus (REACTION), it was found that a higher RC was significantly linked to a greater probability of developing CKD among Chinese individuals aged 40 years and above (29). A recently published report from the REACTION study demonstrated that besides conventional lipids, Non-HDL-C, RC, and various lipid index ratios were more robustly linked to early progression of renal injury (49). Given the established association between renal function and hypertension, as well as the robust correlation observed between the RC index and renal function, our working hypothesis suggests that renal function could potentially serve as a mediating factor in the relationship between RC and hypertension. Our study results indicated that the link between RC and hypertension may be mediated by reduced renal function. Although eGFR only mediated 6% of the association, this result does not imply that the pathway is meaningless; rather, it suggests that there are more complex mechanisms between RC and hypertension. This finding provides a basis for subsequent studies to explore new biological mechanisms. Furthermore, the combined evaluation of RC and eGFR has the potential to provide valuable insights and stratify individuals within the population who are at the greatest risk for hypertension.

Our study also delved into the potential interactions between RC and eGFR related to hypertension risk. Contrary to our initial hypothesis, our findings suggest that there is no significant synergy or interaction between these two factors in influencing hypertension risk. In essence, although both RC and eGFR contribute independently to hypertension risk (18, 20, 45–47), their combined effect does not appear to exceed the sum of their individual effects. This observation complicates our understanding of how RC and eGFR together affect hypertension risk. Hypertension is a multifactorial disease caused by a variety of intertwined risk factors, including age, gender, smoking, alcohol consumption, obesity, genetics and other metabolic diseases (50, 51). The presence of these different factors may moderate the interaction between RC and eGFR, which may explain the lack of significant synergistic effects. Another possibility is that RC and eGFR act through different pathways associated with hypertension risk. Although both factors are associated with metabolic dysfunction, their respective mechanisms may be different.

Although our study did not reveal a synergistic effect between RC and eGFR, it does emphasize the importance of jointly considering these factors in hypertension risk assessment. Our findings suggest that the combined effect of RC and eGFR may be more effective than either factor alone in predicting hypertension risk. The concept of a joint effect implies that individuals with elevated RC and reduced eGFR values may be at higher risk of hypertension than individuals with abnormal values for only one of these variables.

The findings of the present study suggest that the effects of RC and eGFR on moderate to high blood pressure may be linear. CKD often occurs alongside cardiovascular risk factors, including dyslipidemia and hypertension (52). There is evidence that elevated RC levels may lead to oxidative stress and damage endothelial cells (53, 54), which prevents them from properly releasing vasodilators such as nitric oxide, which may lead to vasoconstriction and increased blood pressure. High cholesterol levels can damage the glomeruli. When the glomeruli are damaged, the kidneys’ filtration function is weakened, leading to fluid retention and sodium activation in the body, which in turn causes hypertension (55). However, the exact biological mechanism of their combined action is still unknown.

Our research is subject to certain limitations. First, as our study was observational in nature, we were unable to confirm a causal relationship between RC, renal function, and hypertension as a definitive conclusion. Second, despite our efforts to account for various potential confounding factors, it is important to acknowledge that there may still be unidentified confounders that could impact the results. Third, CHARLS lacked data on urinary protein, renal imaging, and neuropathology, which may have biased the statistical results. Fourth, selection bias may result from excessive missing data at follow-up.

## Conclusion

In the middle-aged and older Chinese demographic, there exists a correlation between RC, renal function, and hypertension. The results of this investigation revealed that impaired kidney function played a pivotal role in mediating the link between RC and hypertension. Therefore, long-term monitoring of RC levels and renal function may help prevent hypertension.

## Data Availability

Publicly available datasets were analyzed in this study. This data can be found here: https://charls.charlsdata.com/index/zh-cn.html.
