# Supplementary material for: Combined and mediating effects of remnant cholesterol and renal function on hypertension risk in Chinese middle-aged and elderly people
Source: Front Endocrinol (Lausanne). 2025 Feb 13;16:1442918. doi: 10.3389/fendo.2025.1442918 (PMC11864924; doi:10.3389/fendo.2025.1442918)
Supplement: Supplementary file 1 [file DataSheet1.docx]

**Supplementary Table 1** Sensitivity analysis after deletion of missing values.

| **Sensitivity analysis** | **Crude** | **P value** | **Model 1** | **P value** | **Model 2** | **P value** | **Model 3** | **P value** |
| --- | --- | --- | --- | --- | --- | --- | --- | --- |
|  | **HR (95%CI)** |  | **HR (95%CI)** |  | **HR (95%CI)** |  | **HR (95%CI)** |  |
| **RC < 18.94 & eGFR ≥ 60** | 1(Ref) |  | 1(Ref) |  | 1(Ref) |  | 1(Ref) |  |
| **RC < 18.94 & eGFR < 60** | 1.60 (0.66,3.87) | 0.299 | 1.75 (0.72,4.26) | 0.217 | 1.95 (0.80,4.77) | 0.145 | 2.07 (0.84,5.08) | 0.112 |
| **RC ≥ 18.94 & eGFR ≥ 60** | 1.23 (1.04,1.45) | 0.013 | 1.21 (1.03,1.43) | 0.021 | 1.11 (0.93,1.32) | 0.238 | 1.10 (0.92,1.31) | 0.305 |
| **RC ≥ 18.94 & eGFR < 60** | 2.83 (1.51,5.33) | 0.001 | 2.86 (1.52,5.39) | 0.001 | 2.90 (1.53,5.50) | 0.001 | 2.88 (1.52,5.47) | 0.001 |

HR, hazard ratio; CI, confidence interval; RC, remnant cholesterol; eGFR, estimated glomerular filtration rate;

We adjusted for potential confounders, including age, sex, education level, marital status, BMI, dyslipidemia, heart disease, stroke, smoking status, drinking status, lipid-lowering drugs, hypoglycemic drug, HbA1c, and CRP.

**Supplementary Table 2** Effect of RC and eGFR with risk of hypertension when analyzed using 3*3 matrix.

| **Sensitivity analysis** | **Crude** | **P value** | **Model 1** | **P value** | **Model 2** | **P value** | **Model 3** | **P value** |
| --- | --- | --- | --- | --- | --- | --- | --- | --- |
|  | **HR (95%CI)** |  | **HR (95%CI)** |  | **HR (95%CI)** |  | **HR (95%CI)** |  |
| RC tertile 1 & eGFR ≥90 | 1(Ref) |  | 1(Ref) |  | 1(Ref) |  | 1(Ref) |  |
| RC tertile 1 & eGFR 60-90 | 1.12 (0.83,1.52) | 0.449 | 1.12 (0.83,1.52) | 0.459 | 1.08 (0.80,1.47) | 0.621 | 1.07 (0.79,1.46) | 0.650 |
| RC tertile 1 & eGFR <60 | 1.95 (0.87,4.39) | 0.107 | 1.83 (0.81,4.15) | 0.145 | 1.95 (0.86,4.42) | 0.109 | 1.97 (0.87,4.47) | 0.105 |
| RC tertile 2 & eGFR ≥90 | 1.06 (0.88,1.28) | 0.512 | 1.05 (0.88,1.27) | 0.582 | 1.00 (0.83,1.21) | 0.984 | 1.00 (0.83,1.20) | 0.969 |
| RC tertile 2 & eGFR 60-90 | 1.14 (0.86,1.52) | 0.363 | 1.14 (0.85,1.51) | 0.384 | 1.07 (0.80,1.43) | 0.633 | 1.07 (0.80,1.43) | 0.634 |
| RC tertile 2 & eGFR <60 | 1.93 (0.91,4.11) | 0.086 | 1.94 (0.91,4.12) | 0.086 | 1.88 (0.88,4.00) | 0.102 | 1.89 (0.89,4.02) | 0.100 |
| RC tertile 3 & eGFR ≥90 | 1.44 (1.21,1.72) | <0.001 | 1.44 (1.21,1.72) | <0.001 | 1.23 (1.02,1.48) | 0.033 | 1.21 (1.00,1.46) | 0.045 |
| RC tertile 3 & eGFR 60-90 | 1.59 (1.25,2.04) | <0.001 | 1.60 (1.25,2.04) | <0.001 | 1.33 (1.03,1.72) | 0.030 | 1.32 (1.02,1.71) | 0.033 |
| RC tertile 3 & eGFR <60 | 2.33 (1.24,4.40) | 0.009 | 2.25 (1.19,4.24) | 0.012 | 1.97 (1.04,3.74) | 0.037 | 1.97 (1.04,3.73) | 0.039 |

HR, hazard ratio; CI, confidence interval; RC, remnant cholesterol; eGFR, estimated glomerular filtration rate;

eGFR was calculated using CKD-EPI (Chronic Kidney Disease Epidemiology Collaboration) equation; unit of eGFR: ml/minute/1.73 m^2^; RC was grouped by tertile; eGFR was grouped by 60 and 90 ml/minute/1.73 m^2^

We adjusted for potential confounders, including age, sex, education level, marital status, BMI, dyslipidemia, heart disease, stroke, smoking status, drinking status, lipid-lowering drugs, hypoglycemic drug, HbA1c, and CRP.


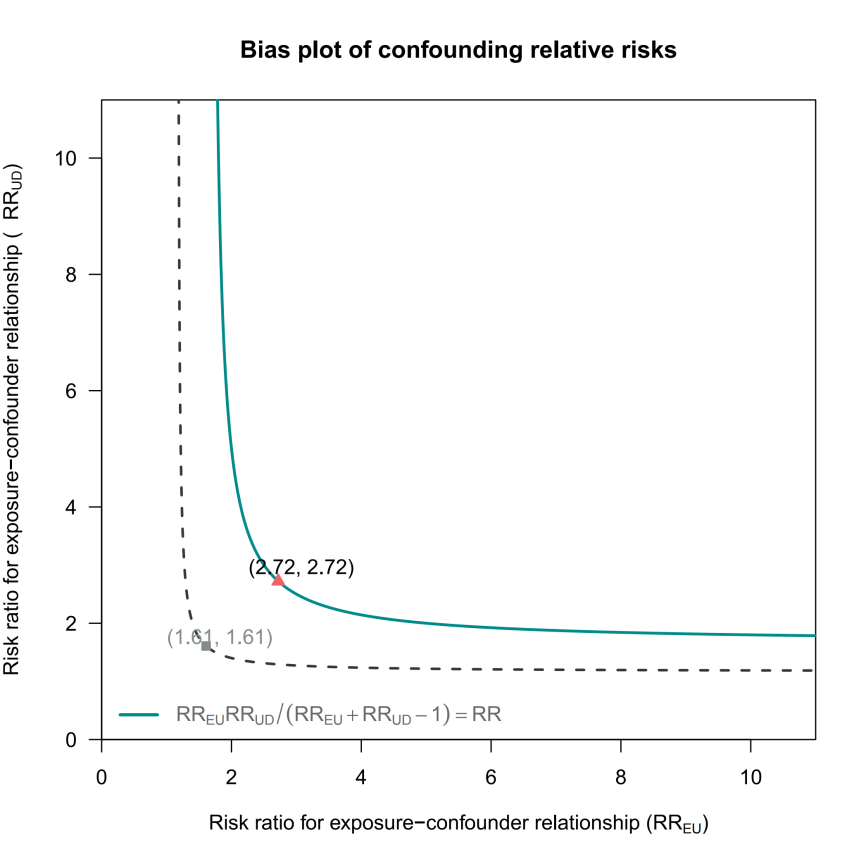


**Supplementary Figure 1** Curves of the sensitivity analysis for unobserved confounders with E-value highlighted.

Curve depicting the range of joint relationships (RC and eGFR-confounder and confounder-hypertension) that may explain away the estimated effect and its confidence interval for the multivariable Cox regression model to predict hypertension.


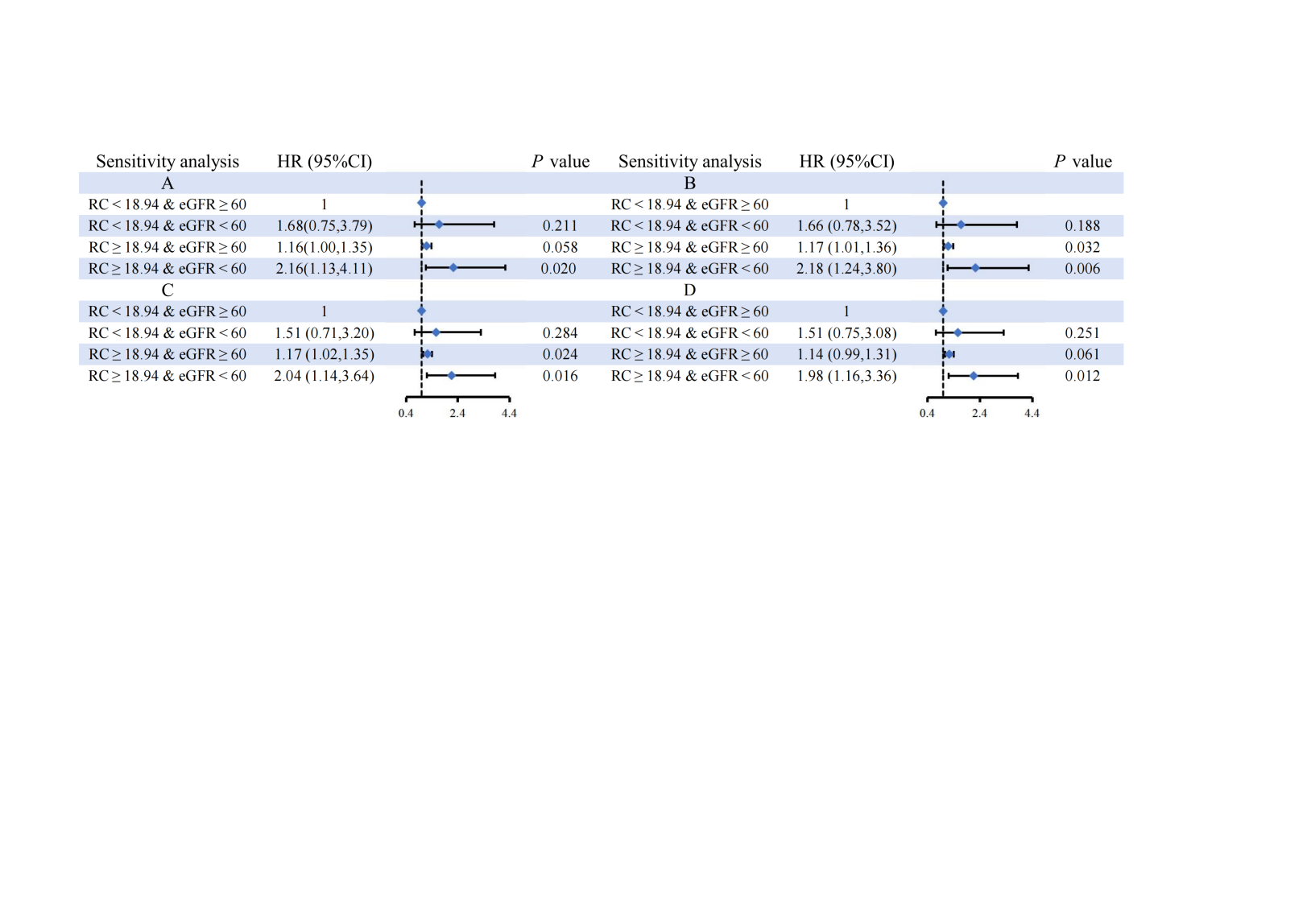


**Supplementary Figure 2** Sensitivity analyses for the combined assessment of RC and eGFR levels and risk of hypertension.

We adjusted for potential confounders, including age, sex, education level, marital status, BMI, dyslipidemia, heart disease, stroke, smoking status, drinking statuslipid-lowering drugs, hypoglycemic drug, HbA1c, and CRP.

Sensitivity analysis A: model construction using IPTW; sensitivity analysis B: exclusion of those with cardiovascular disease; sensitivity analysis C: exclusion of those on lipid-lowering and glucose-lowering medications; sensitivity analysis D: additional adjustment for UA levels.


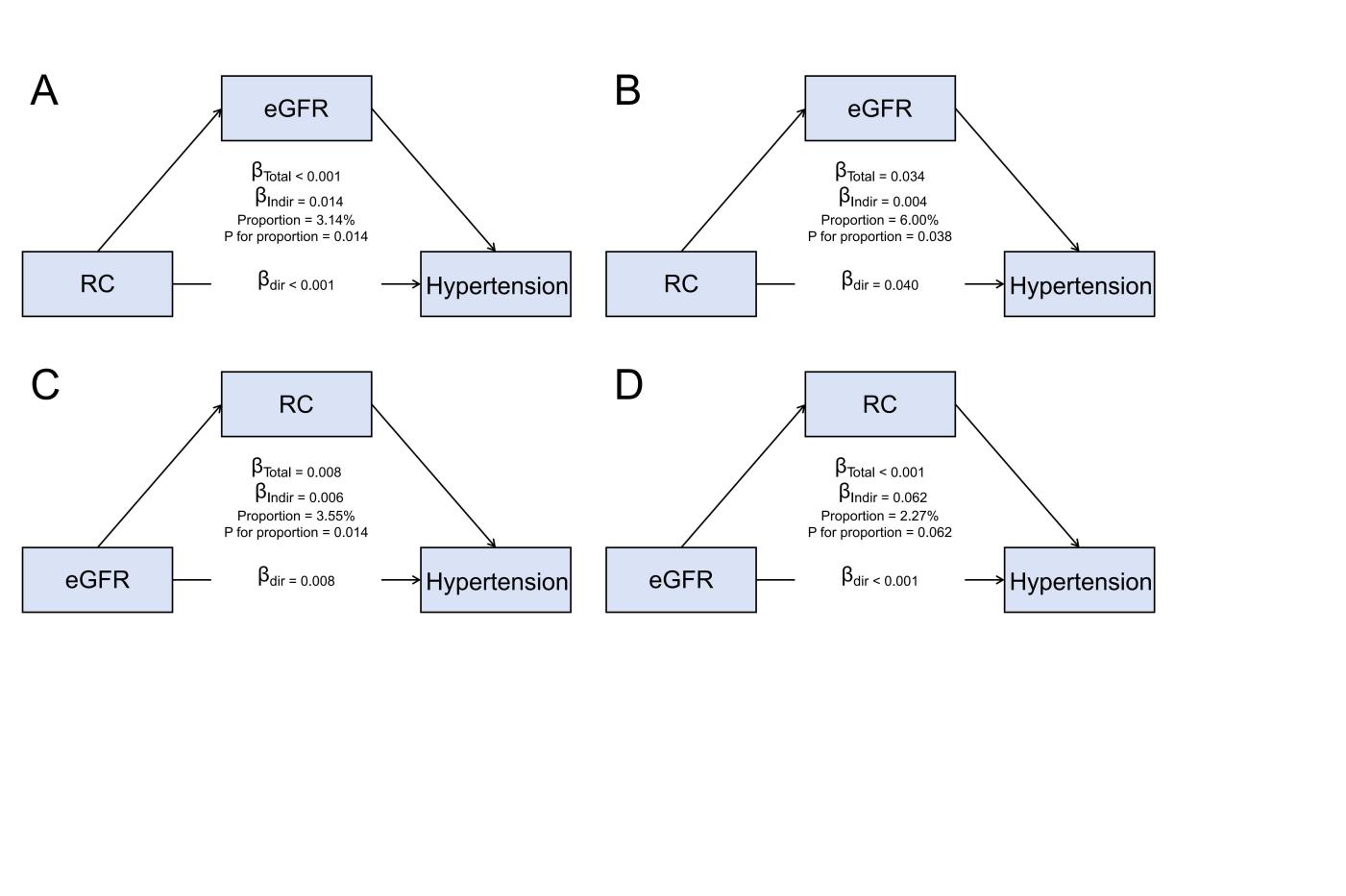


**Supplementary Figure 3** Mediating role analysis. (A) Unadjusted eGFR mediation; (B) Mediation analysis of eGFR after adjusting for age, sex, education level, marital status, BMI, dyslipidemia, heart disease, stroke, smoking status, drinking statuslipid-lowering drugs, hypoglycemic drug, HbA1c, and CRP; (C) Unadjusted RC mediation; (D) Mediation analysis of RC after adjusting for age, sex, education level, marital status, BMI, dyslipidemia, heart disease, stroke, smoking status, drinking statuslipid-lowering drugs, hypoglycemic drug, HbA1c, and CRP.
